# Coagulase gene polymorphism, enterotoxigenecity, biofilm production, and antibiotic resistance in *Staphylococcus aureus* isolated from bovine raw milk in North West India

**DOI:** 10.1186/s12941-017-0242-9

**Published:** 2017-09-20

**Authors:** Vishnu Sharma, Sanjita Sharma, Dinesh Kumar Dahiya, Aarif Khan, Manisha Mathur, Aayushi Sharma

**Affiliations:** 1 Advanced Milk Testing Research Laboratory, Postgraduate Institute of Veterinary Education and Research, Rajasthan University of Veterinary and Animal Sciences at Bikaner, B-2 Bypass, Shiprapath, Mansarovar, Jaipur, 302020 Rajasthan India; 2 Sawai Man Singh Medical College, Jaipur, 302004 Rajasthan India

**Keywords:** *Staphylococcus aureus*, Coagulase gene polymorphism, RFLP, Biofilm, Enterotoxin, Antibiotic susceptibility

## Abstract

**Background:**

*Staphylococcus aureus* is the predominant bacterium responsible for various diseases in animals and humans. Preventive strategies could be better implemented by understanding the prevalence, genetic patterns, and the presence of enterotoxin and biofilm-producing genes along with the antibiotic susceptibility of this organism. This study was conducted in Rajasthan, the northwestern state of India, holding the largest population of cattle that makes it the second largest milk producer in India and no such prior information is available on these aspects.

**Methods:**

A total of 368 individual quarter bovine raw milk samples were collected from 13 districts of Rajasthan, and screened for the presence of *S. aureus*. Microbiological and molecular approaches were followed for bacterial identification. Genetic diversity was determined by polymerase chain reaction-restriction fragment length polymorphism (PCR–RFLP) of coagulase gene (*coa*), whereas enterotoxin and biofilm-producing genes were studied by PCR analysis. Antibiotic strips were employed to study the antibiotic resistance among strains.

**Results:**

In all, 73 *S. aureus* strains were obtained from 368 bovine raw milk samples out of that only 30 showed the presence of *coa*. Nine types of *coa* patterns ranging from 730 to 1130 bp were observed among these isolates. PCR–RFLP of *coa* distinguished the isolates into 15 genotypic patterns, of which patterns I, IV, V, and VI were predominant. Of the isolates, 30% were positive for *sec*, 10% for *sea*, and 3.3% for *seb*; these genes are responsible for enterotoxin production, whereas all isolates were found positive for *ica*AD and *eno*. The prevalence rates of other biofilm-producing genes *fnb*A, *clf*B, *ebp*S, *sas*G, *fnbB, sas*C, *cna*, *bap*, *fib* and, *bbp* were 97, 93, 90, 80, 80, 77, 53, 27, 10, and 6.6%, respectively. Twenty-seven (90%) strains were multidrug resistant, of which 15 were methicillin resistant. Maximum sensitivity was reported for kanamycin and it could be considered as a drug of choice for controlling *S. aureus* mediated cattle infections in the studied regions.

**Conclusions:**

Overall, these strains could cause several diseases to humans, insisting the need for developing a stricter hygiene program for improving milking practices and animal health.

## Background

The dairy sector in India has grown substantially over the years, making India the world’s largest producer of milk [1], and Rajasthan, the northwestern state of India, has the largest number of cattle that makes it the second largest milk producer in India [2]. However, the yield of animals within the state is not satisfactory due to various production diseases and is understood as the major factor responsible for economic losses to the dairy industry in India [3, 4].

Subclinical mastitis (SCM) with no sign of inflammation is more severe than clinical forms and is responsible for a huge loss to the dairy industry [5, 6]. *Staphylococcus aureus* is a part of the normal gut microbiota [7] but is also a predominant microorganism implicated in clinical mastitis and SCM [8] and is difficult to eliminate. The pathogenic strains of *S. aureus* are generally coagulase positive and reported to cause illness in their host around the world [9]. From the infected udders, *S. aureus* directly passes into the raw milk and affects the quality of milk and makes the milk less suitable for further product formulations (e.g., cheese, paneer [a soft, white cheese]). In India, 85% of total milk produced is handled by the noncommercial sector [1]. Therefore, the direct consumption and transformation of such milk into traditional dairy foods increase the chances of associated infections because of improper storage and transportation facilities. The hygiene present in dairies and the literacy of handlers about safe milk production in Rajasthan are below satisfactory level [10]. For the effective control measures of *S. aureus*, its genetic characterization is necessary [11, 12]. Coagulase gene (*coa*) typing has been proven to be the most successful method to discriminate isolates at the strain level, recovered from different regions, mainly because of simple, accurate, and reproducible results obtained with this technique over other methods [13–15].

Multiple virulence factors have been suspected for the pathogenicity of *S. aureus*, among which the production of enterotoxins and toxic shock syndrome toxin–1 and the ability to form biofilms are important and worrisome for the food industry [16, 17]. Staphylococcal enterotoxins (SEs) are heat-stable; therefore, they may retain their biological activity even after pasteurization and various processing steps. An accidental ingestion of SEs causes several gastrointestinal disorders in the host [18].

Biofilm-producing *S. aureus* strains, especially the enterotoxigenic and antibiotic-resistant ones, are the major cause of several persistent bacterial diseases in the livestock sector, including mastitis [19]. During biofilm formation, initially, the bacteria adhere to each other by polysaccharide intercellular adhesion (PIA) and then propagate. The PIA is under the control of the *icaADBC* operon, and the strains possessing this gene cluster have been reported as strong biofilm producers [20]. In addition, several other surface markers have been reported to play a crucial role in biofilm formation, specifically called as microbial surface components recognizing adhesive matrix molecules (MSCRAMMs) such as collagen-binding protein (Cna) and elastin-binding protein (Ebps). The formation of biofilm also provides a shielding effect to the bacterium from antibiotic attacks.

To tackle *S. aureus*-mediated infections, antibiotics are used by veterinary professionals but their therapeutic outcomes are nearly insignificant because of the stubborn nature of the pathogen [21]. The frequent use of antibiotics leads to the evolution of antibiotic-resistance genes in *S. aureus* that can be easily transferred among healthier commensals and to other animals and humans by close interactions [9]. Studying the response of *S. aureus* to different relevant antibiotics gives the present drug resistance scenario from a region and the measure of associated risk factors. In addition, it may also help in selecting a more effective drug from a veterinary clinical point of view.

Therefore, the aims of this study were to check the prevalence of *S. aureus* from bovine raw milk samples and determine the genetic heterogeneity, virulence potential, and antimicrobial susceptibility of *S. aureus* from different regions of Rajasthan. These tasks were performed to generate useful information for field veterinarians and policy makers to form strategies for controlling *S. aureus*-mediated infections and ultimately improving the quality of milk.

## Methods

### Milk sampling and microbiological analysis

**Fig. 1:**
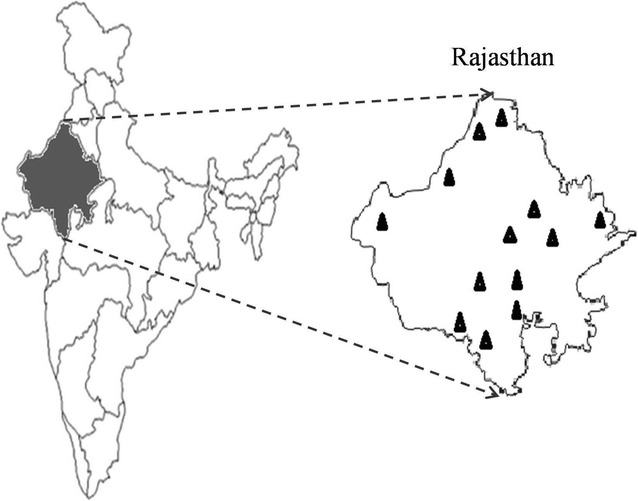
Map of Rajasthan India showing the districts from where sampling was carried out

From May 2014 to November 2015, 368 individual quarter bovine raw milk samples were collected from 13 districts of Rajasthan, India (Fig. 1). The samples were randomly taken from clinically healthy animals. The samples were collected from different dairy herds, and before sampling, the teats of animals were cleaned with cotton balls soaked with 70% alcohol and the first few milliliters of milk were discarded. The samples were maintained at 4 °C before processing in a laboratory. The samples were serially diluted in 2% peptone water, and 100 µL of each sample was poured onto *S. aureus* chromogenic agar plates (HiMedia Laboratories, Mumbai, India) containing polymyxin B (50 unit/mL). After 24-h of incubation at 37 °C, *Staphylococcus* colonies showing blue-green color from each sample were transferred to brain heart infusion (BHI) broth. The chromogenic mixture present in the medium is specifically cleaved by *S. aureus* to produce blue-green colonies, which are clearly visible against the opaque background. For reconfirmation, the cultures were streaked on Mannitol salt agar and Baird Parker agar plates (Himedia Laboratories). Next, the colonies were examined for morphology after Gram staining and were confirmed using the API Staph kit (bioMeriux, Marcy-l’Etoile, France). Confirmed *S. aureus* colonies were examined for coagulase activity by tube plasma agglutination test. The presumptively identified *S. aureus* colonies were maintained at −80 °C in glycerol stocks for further analysis. A single colony was picked per positive sample and used to determine the prevalence rate among different samples. The isolates were coded conforming to their origin.

### DNA extraction and purification

Genomic DNA was extracted from a freshly grown culture in BHI broth by using a modified method [22]. Briefly, 2 mL of overnight grown culture was centrifuged at 12,000×*g* for 10 min to form pellets. Afterwards, the pellets were resuspended in 200 µL of sucrose EDTA Tris (SET) buffer containing lysostaphin (100 µg/mL; Sigma, St. Louis, Missouri, USA) and lysozyme (100 µg/mL; Amersco, Solon, USA) for 1 h at 37 °C. The remaining procedure was similar as described elsewhere [22]. The extracted DNA was examined for purity and concentration using a microplate reader at 260 nm and thereafter stored at −20 °C.

All conventionally identified *S. aureus* were ascertained by species-specific primers (F 5′-CCATGTGTAGCGGTGAAATG–3′ and R 5′-TAAGGTTCTTCGCGTTGCTT–3′) obtained from the 16S rRNA region of *S. aureus* (GenBank accession number: KF993676.1) by using Primer 3 plus software. A 25-µL PCR reaction mixture consisting of 12.5 µL of 2X DreamTaq Green PCR Master Mix (Thermo Scientific, Mumbai, India), 0.5 µM of each primer, and 1 µL of template DNA was prepared and performed in a SureCycler 8800 Thermal Cycler (Agilent Technologies, New Delhi, India). Following program was used for PCR cycles: 2 min at 95 °C, followed by 35 cycles of 30 s at 95 °C, 45 s at 57 °C, and 60 s at 72 °C with a final extension of 10 min at 72 °C. A PCR product of approximately 306 bp signifies the presence of *S. aureus* isolates.

Using an earlier protocol, *coa* was identified in the *S. aureus* isolates [23]. The amplified products were electrophoresed on 2.0% agarose gel at 100 V for 1 h and photographed in a gel documentation system (BioRad Laboratories, Gurgaon, India). A suitable ladder was used as the size marker during electrophoresis.

### Restriction enzyme digestion of PCR-amplified *coa* product

Of the PCR-amplified *coa* product, 10 µL was mixed with 17 µL of nuclease-free water, 2 µL of buffer Tango, and 1 µL of *Alu*I (Thermo Scientific) and incubated at 37 °C for 1 h for digestion. The digested products were resolved on 2.0% agarose gel and examined in a gel documentation system. A 100–1500-bp ladder (Promega, Wisconsin, USA) was used as the size marker for analyzing the digested *coa* fragments.

The generated Coa-RFLP patterns were analyzed by the BioNumerics (version 7.6) software package (Applied Maths, Sint-Martens-Latem, Belgium). A dendrogram was prepared by using the Dice-coefficient and the unweighted pair group method with arithmetic averages as the cluster analysis method.

### Reproducibility testing

PCR reproducibility was analyzed by testing five isolates for five consecutive days. The PCR–RFLP reproducibility of *coa* was determined by digesting four products twice with the *Alu*I enzyme.

### Calculating numerical index of discrimination

A numerical index equation was used to determine the discriminatory power of the typing method as described previously [24]. The following formula was used:

$$D = 1 - \frac{1}{N(N - 1)}\mathop \sum \limits_{j = 1}^{s} nj(nj - 1)$$

where *D* = discriminatory index, *s* = total number of different types, *nj* = number of isolates representing each type, and *N* = total number of isolates in the sample population.

### Detection of enterotoxin and biofilm genes

The amplification of the genes coding for enterotoxins and biofilm production (Table 1) was performed using the multiplex PCR method [25, 26].

**Table 1:** Primers used for detection of *S. aureus* enterotoxin and biofilm genes

Gene	Primer sequence (5′–3′)	Product size (bp)	Multiplex PCR set
*sea* ^*a*^	GGTTATCAATGTGCGGGTGG CGGCACTTTTTTCTCTTCGG	102	A
*seb* ^*a*^	GTATGGTGGTGTAACTGAGC CCAAATAGTGACGAGTTAGG	164	A
*sec* ^*a*^	AGATGAAGTAGTTGATGTGTATGG CACACTTTTAGAATCAACCG	451	A
*sed* ^*a*^	CCAATAATAGGAGAAAATAAAAG ATTGGTATTTTTTTTCGTTC	278	A
*see* ^*a*^	AGGTTTTTTCACAGGTCATCC CTTTTTTTTCTTCGGTCAATC	209	A
*eta* ^*a*^	GCAGGTGTTGATTTAGCATT AGATGTCCCTATTTTTGCTG	93	B
*etb* ^*a*^	ACAAGCAAAAGAATACAGCG GTTTTTGGCTGCTTCTCTTG	226	B
*tst* ^*a*^	ACCCCTGTTCCCTTATCATC TTTTCAGTATTTGTAACGCC	326	B
*bap* ^*b*^	GAGCCAAGACAAAGGTGAAG GTAGCCATAGCACGGAACAT	873	
*bbp* ^*b*^	CTTAGCAGTTCAACAGGGTG TTGGCTTTATTGTGATGGTC	1662	
*cna* ^*b*^	CGATAACATCTGGGAATAAA ATAGTCTCCACTAGGCAACG	716	
*clfA* ^*b*^	AGTACCAAATGAGGCTGTTC AAATGCTACTTCGTTGTCCC	796	
*clfB* ^*b*^	CACTTACTTTACCGCTACTTTC AACGAGCAATACCACTACAACAG	968	
*ebpS* ^*b*^	GGTGAACCTGAACCGTAG CTGGCAAGGCGAATAACT	661	
*eno* ^*b*^	GTCGTGCATTAGTACCATCAGG TTTCCAACCATCCCAGTCG	812	
*fib* ^*b*^	AGATGCGAGCGAAGGGTA TAAACGAAACTAAGTTGACTGC	347	
*fnbpA* ^*b*^	TCCGCCGAACAACATACC TCAAGCACAAGGACCAAT	952	
*fnbpB* ^*b*^	TCTGCGTTATGAGGATTT ACAGTAGAGGAAAGTGGG	452	
*icaAD* ^*b*^	TGGCTACTGGGATACTGATA TGGAAATGCGACAAGAACTA	520	
*icaBC* ^*b*^	GCCTATCCTTATGGCTTGA TGGAATCCGTCCCATCTC	182	
*sasC* ^*b*^	AGAATGAAGTCCGATAGAGT AATCATACAGATGGCAATAC	936	
*sasG* ^*b*^	TATCAACACTTCCGTAACCTTC CGTCAGTCACTCATAACGCAGA	159	

^a^Represents the enterotoxin genes while ^b^ depicts the biofilm genes

### Determination of minimum inhibitory concentration

The minimum inhibitory concentration (MIC) of all *S. aureus* isolates was determined using E-strips as per the manufacturer’s instructions after adjusting their OD to McFarland 0.5 as disclosed previously [27]. The results obtained were analyzed according to CLSI guidelines [28]. The MIC E-strips (0.016–256 µg/mL) (HiMedia Laboratories) were used for susceptibility observation. The antimicrobials used were according to the information collected from the field veterinarians and practicing clinicians. *S. aureus* ATCC 29213 was used as the reference strain for quality control. Methicillin-resistant *S. aureus* (MRSA)-positive strains were further confirmed on HiCrome MeReSa Agar (HiMedia Laboratories) and validated by the amplification of *mec*A by PCR [29].

## Results

**Fig. 2:**
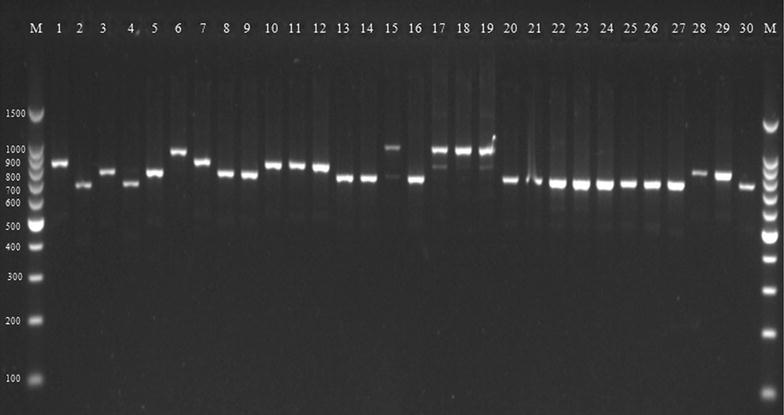
Agarose gel electrophoresis of the coagulase gene (*coa*). Lane M; 100–1500 bp molecular marker, 1-STRMCOR7 (920 bp), 2-STRMPL47(730 bp), 3-STRMSH56 (820 bp), 4-STRMBHL57 (750 bp), 5-STRMSH58 (820 bp), 6-STRMHMO59 (1060 bp), 7-STRMUDZ70 (920 bp), 8-STRMSH73 (820 bp), 9-STRMSH74 (820 bp), 10-STRMHMO79 (920 bp), 11-STRMSH37 (920 bp), 12-STRMSH42 (920p), 13-STRMMA11 (820 bp), 14-STRMMA20 (820p), 15-STRMUDZ2 (1130 bp) 16-STRMMA3 (820 bp), 17-STRMSGNR64 (1130 bp), 18-STRMSGNR19 (1130 bp), 19-STRMUDZ6 (1130 bp), 20-STRMBKN5 (820 bp), 21-STRMSK96 (820 bp), 22-STRMSK98 (820 bp), 23-STRMSK100 (820 bp), 24-STRMBP11 (820 bp), 25-STRMB12 (820 bp), 26-STRMJ102 (820 bp), 27-STRMJ3 (820 bp), 28-STRMP23 (920 bp), 29-STRMSGNR9 (880 bp), 30-STAJM33 (800 bp)

**Fig. 3:**
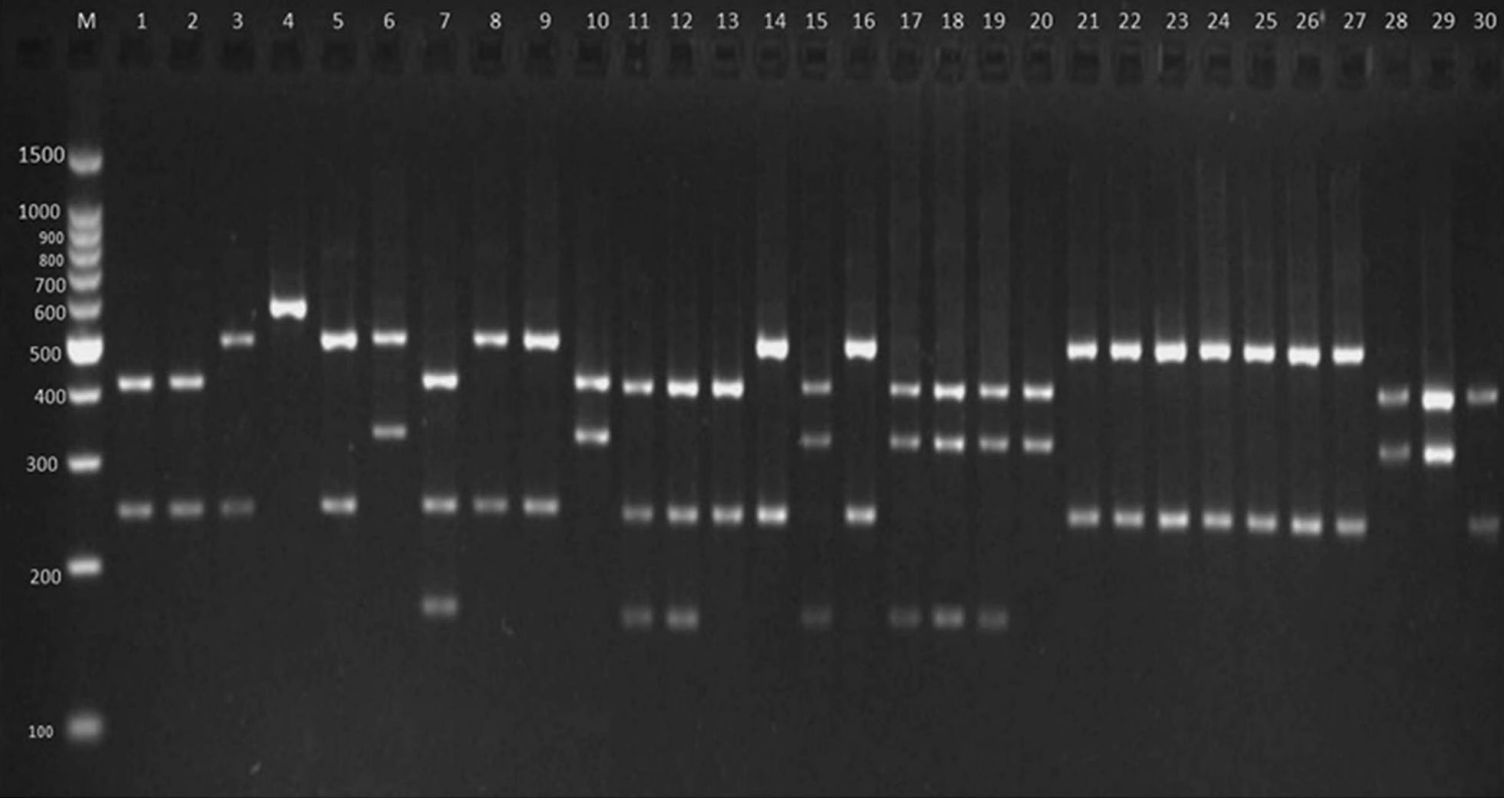
RFLP of the coagulase gene (coa) after digestion with *Alu*I. Lane M; 100–1500 bp molecular marker, 1-STRMCOR7, 2-STRMPL47, 3-STRMSH56, 4-STRMBHL57, 5-STRMSH58, 6-STRMHMO59, 7-STRMUDZ70, 8-STRMSH73, 9-STRMSH74, 10-STRMHMO79, 11-STRMSH37, 12-STRMSH42, 13-STRMMA11, 14-STRMMA20, 15-STRMUDZ2, 16-STRMMA3, 17-STRMSGNR64, 18-STRMSGNR19, 19-STRMUDZ6, 20-STRMBKN5, 21-STRMSK96, 22-STRMSK98, 23-STRMSK100, 24-STRMBP11, 25-STRMB12, 26-STRMJ102, 27-STRMJ3, 28-STRMP23, 29-STRMSGNR9, 30-STAJM33

**Fig. 4:**
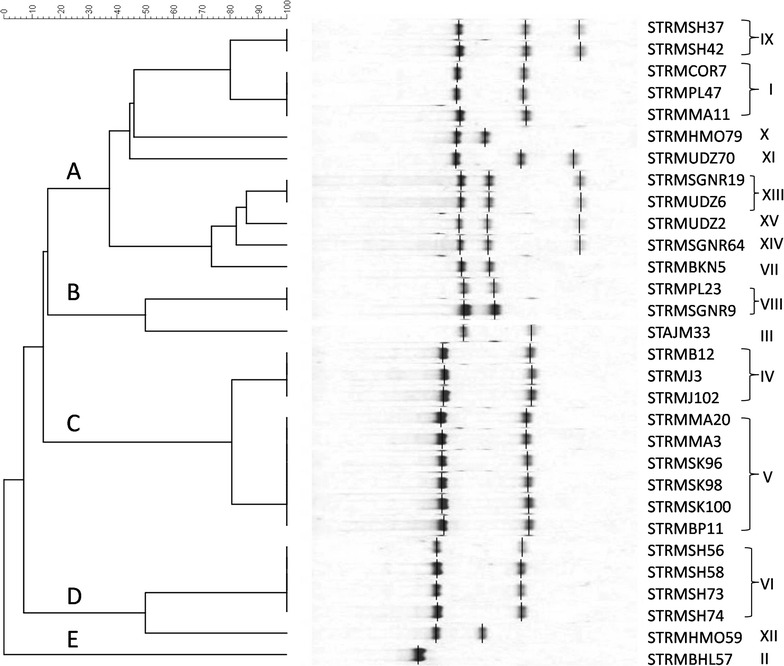
Dendrogram showing genetic heterogeneity among *S. aureus* isolates on the basis of the *coa* gene polymorphism

Out of 368 bovine raw milk samples, *S. aureus* contamination was present in 73 (19.84%) samples (Table 2) as revealed by media plating analysis. All 73 isolates were found as *S. aureus* in API analysis. Out of the 73 isolates, 30 (41.1%) showed coagulase activity and were further confirmed as *S. aureus* by PCR. Furthermore, all these phenotypically coagulase-positive *S. aureus* scored positive for *coa* during PCR analysis (Fig. 2). Nine types of *coa* patterns ranging from 730 to 1130 bp were observed (Table 3), and the sizes, 820 and 920 bp, were the most predominantly noticed product sizes, accounting altogether for 67% of the total *coa*-positive isolates (Table 3). Twenty-seven isolates depicted one PCR band while three isolates (15, 17, and 19) depicted double bands (Fig. 2). The polymorphism in *coa* was studied by RFLP (Fig. 3), where fifteen (I–XV) RFLP patterns (Table 3 and Fig. 4) were observed. RFLP patterns I, IV, V, and VI were the dominant patterns being noticed in three (10%), three (10%), six (20%), and four (13.3%) isolates, respectively (Table 3). The isolates with the most predominant pattern (V) displayed two DNA fragments of 540 and 260 bp. The isolates with pattern VI had DNA fragments of 565 and 270 bp. The isolates with patterns I and IV had DNA fragments of 430 and 260 bp and 528 and 250 bp, respectively. Rest all other remaining patterns were shown by a single (II, VII, X, XI, XII, and XIV) or two (VIII, IX, and XIII) isolates. The reproducibility of the PCR products and *coa* PCR–RFLP was demonstrated with 100% of the repeatedly tested isolates; however, the results showed some variations in the intensity of bands.

**Table 2:** Details of samples contaminated with *S. aureus* and their prevalence

Sampling districts/regions	No. of raw milk samples collected	No. of positive samples (% prevalence)
Ajmer	28	4 (1.09)
Bharatpur	28	7 (1.90)
Bhilwara	28	3 (0.82)
Bikaner	28	2 (0.54)
Chittorgarh	28	4 (1.09)
Hanumangarh	28	7 (1.90)
Jaipur	30	5 (1.36)
Mount Abu	28	8 (2.17)
Pali	28	6 (1.63)
Sirohi	30	13 (3.53)
Sikar	28	5 (1.36)
Shri-Ganganagar	28	6 (1.63)
Udaipur	28	3 (0.82)
Total	368	73 (19.84)

**Table 3:** Genotype distributions of *S. aureus* cultures obtained from different regions of Rajasthan and their RFLP patterns

*Coa* gene PCR product (bp)	RFLP pattern	n	Isolate name	Isolated region	Genotype code/distinct RFLP pattern
730	430-260	1	STRMPL47	PL; Pali	I
750	620	1	STRMBHL57	BHL; Bhilwara	II
800	420-240	1	STAJM33	AJM; Ajmer	III
820	528-250	3	STRMBP12, STRMJ102, STRMJ3	BP; Bharatpur, J; Jaipur	IV
820	540-260	6	STRMMA20, STRMMA3, STRMSK96, STRMSK 98, STRMSK100, STRMBP11	SH; Sirohi MA; Mount abu, SK; Sikar, BP; Bharatpur	V
820	565-270	4	STRMSH56, STRMSH58, STRMSH73, STRMSH74	SH; Sirohi	VI
820	430-260	1	STRMMA11	MA; Mount abu	I
820	440-360	1	STRMBKN5	BKN; Bikaner	VII
880	420-330	1	STRMSGNR9	SGNR; Shri-Ganganagar	VIII
920	440-260-170	2	STRMSH37, STRMSH42	SH; Sirohi	IX
920	420-330	1	STRMPL23	PL: Pali	VIII
920	462-373	1	STRMHMO79	HMO; Hanumangarh	X
920	425-260	1	STRMCOR7	COR; Chittorgarh	I
940	452-273-186	1	STRMUDZ70	UDZ; Udaipur	XI
1060	540-350	1	STRMHMO59	HMO; Hanumangarh	XII
1130	445-360-170	2	STRMSGNR19, STRMUDZ6	SGNR; Shri-Ganganagar, UDZ; Udaipur	XIII
1130	452-370-175	1	STRMSGNR64	SGNR; Shri-Ganganagar	XIV
1130	460-380-182	1	STRMUDZ2	UDZ; Udaipur	XV

The discriminatory index for the *Alu*I RFLP method was about 0.80, indicating a fair discriminatory power of this method for typing *S. aureus*. Further RFLP analysis classified the 30 *S. aureus* strains into five major clusters designated as A, B, C, D, and E (Fig. 4). The dendrogram depicted that cluster A was 40% similar to other clusters (B-D) and was composed of isolates obtained from eight regions. Cluster B was 52% identical with clusters C to E and was composed of isolates obtained from three regions. Similarly, cluster C with approximately 82% identity with clusters D and E included isolates from four regions. Cluster D was composed of five isolates obtained from two regions. The remaining cluster E was composed of only one isolate that was derived from a totally different region.

Of the analyzed enterotoxin genes, 30% isolates were positive for *sec*, 10% for *sea*, and 3.3% for *seb* (Table 4). No isolate was detected harboring the remaining enterotoxin genes.

**Table 4:** Results of enterotoxins genes in *S. aureus* isolates

Isolates (n = 30)	*sea*	*seb*	*sec*	*sed*	*see*	*eta*	*etb*	*tst*
STRMPL47	–	+	–	–	–	–	–	–
STRMPL23	–	–	–	–	–	–	–	–
STRMBHL57	+	–	+	–	–	–	–	–
STAJM33	–	–	–	–	–	–	–	–
STRMSH56	–	–	+	–	–	–	–	–
STRMSH58	–	–	–	–	–	–	–	–
STRMSH73	–	–	–	–	–	–	–	–
STRMSH74	–	–	+	–	–	–	–	–
STRMSH37	–	–	–	–	–	–	–	–
STRMSH42	–	–	–	–	–	–	–	–
STRMMA20	–	–	–	–	–	–	–	–
STRMMA3	–	–	–	–	–	–	–	–
STRMMA11	–	–	+	–	–	–	–	–
STRMSK96	–	–	–	–	–	–	–	–
STRMSK 98	–	–	–	–	–	–	–	–
STRMSK100	–	–	–	–	–	–	–	–
STRMBP11	+	–	+	–	–	–	–	–
STRMBP12	–	–	–	–	–	–	–	–
STRMJ102	–	–	–	–	–	–	–	–
STRMJ3	–	–	–	–	–	–	–	–
STRMBKN5	–	–	+	–	–	–	–	–
STRMSGNR9	–	–	–	–	–	–	–	–
STRMSGNR19	–	–	–	–	–	–	–	–
STRMSGNR64	+	–	+	–	–	–	–	–
STRMCOR7	–	–	–	–	–	–	–	–
STRMHMO79	–	–	–	–	–	–	–	–
STRMHMO59	–	–	+	–	–	–	–	–
STRMUDZ70	–	–	+	–	–	–	–	–
STRMUDZ2	–	–	–	–	–	–	–	–
STRMUDZ6	–	–	–	–	–	–	–	–
Occurrence (%)	10	3.3	30	0	0	0	0	0

Regarding biofilm-encoding genes, all isolates typed 100% positive for *ica*AD, *ica*BC, and *eno* (Table 5). The prevalence rates of *fnb*A, *clf*B, *ebp*S, *sas*G, *fnbB, sas*C, *cna*, *bap*, *fib,* and *bbp* were 97, 93, 90, 80, 80, 53, 77, 27, 10, and 6.6%, respectively. Low prevalence was noticed for *clf*A (3.3%) as only one isolate was found positive for this gene.

**Table 5:** Results of adhesion genes typed in *S. aureus* isolates

Isolates (n = 30)	*ica*AD	*ica*BC	*clf*B	*clf*A	*fnb*A	*fnb*B	*ebp*S	*eno*	*fib*	*sas*G	*sas*C	*bbp*	*bap*	*cna*
STRMPL47	+	+	+	–	+	+	+	+	–	–	–	–	–	–
STRMPL23	+	+	+	–	+	+	+	+	–	+	–	–	–	+
STRMBHL57	+	+	+	–	+	+	+	+	+	+	+	+	+	+
STAJM33	+	+	+	–	+	+	+	+	–	+	–	–	–	–
STRMSH56	+	+	+	–	+	+	+	+	–	+	+	–	–	+
STRMSH58	+	+	+	–	+	+	+	+	–	+	+	–	–	+
STRMSH73	+	+	+	–	+	+	+	+	–	+	–	–	–	+
STRMSH74	+	+	+	–	+	+	+	+	–	+	+	–	+	+
STRMSH37	+	+	+	–	+	+	+	+	–	+	+	–	–	+
STRMSH42	+	+	+	–	+	–	–	+	–	+	–	–	–	–
STRMMA20	+	+	+	–	+	+	+	+	–	+	–	–	–	+
STRMMA3	+	+	+	–	+	–	+	+	–	–	–	–	–	–
STRMMA11	+	+	+	–	+	+	+	+	–	+	+	–	–	+
STRMSK96	+	+	+	–	+	+	+	+	–	+	+	–	–	+
STRMSK 98	+	+	+	–	+	+	+	+	–	+	–	–	–	+
STRMSK100	+	+	+	–	+	+	+	+	–	+	+	–	–	+
STRMBP11	+	+	+	+	+	+	+	+	+	+	+	+	+	+
STRMBP12	+	+	+	–	+	+	+	+	–	+	+	–	+	+
STRMJ102	+	+	–	–	–	–	–	+	–	–	–	–	–	–
STRMJ3	+	+	+	–	+	+	+	+	–	+	+	–	–	+
STRMBKN5	+	+	+	–	+	–	+	+	–	+	–	–	–	+
STRMSGNR9	+	+	+	–	+	+	+	+	+	+	+	–	+	+
STRMSGNR19	+	+	+	–	+	+	+	+	–	+	+	–	+	+
STRMSGNR64	+	+	+	–	+	–	+	+	–	–	–	–	–	+
STRMCOR7	+	+	+	–	+	+	+	+	–	+	+	–	–	+
STRMHMO79	+	+	+	–	+	+	+	+	–	+	+	–	+	+
STRMHMO59	+	+	–	–	+	–	–	+	–	–	–	–	–	–
STRMUDZ70	+	+	+	–	+	+	+	+	–	+	–	–	–	+
STRMUDZ2	+	+	+	–	+	+	+	+	–	+	+	–	+	+
STRMUDZ6	+	+	+	–	+	+	+	+	–	–	–	–	–	–
Occurrence (%)	100	100	93	3.3	97	80	90	100	10	80	53	6.6	27	77

**Fig. 5:**
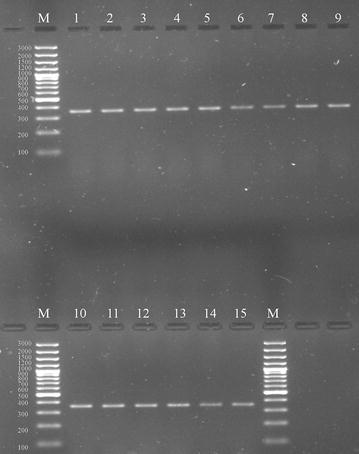
Agarose gel photograph showing the MRSA positive samples. Lane M; 100–3000 bp molecular marker, 1-STRMSH74, 2-STRMSH37, 3-STRMMA20, 4-STRMMA3, 5-STRMMA11, 6-STRMSK98, 7-STRMBP11, 8-STRMBP12, 9-STRMJ3, 10-STRMBKN5, 11-STRMSGNR19, 12-STRMSGNR64, 13-STRMUDZ2, 14-STRMUDZ2, 15-STRMUDZ6

Antibiotic susceptibility test revealed that 27 (90%) isolates were resistant to penicillin, 23 (77%) to ampicillin, 17 (57%) to amikacin, 15 (50%) to oxacillin, 12 (40%) to ciprofloxacin, 9 (30%) to azithromycin, and 7 (23%) to piperacillin (Table 6). Five (17%) isolates showed resistance to linezolid and tetracycline; 3 (10%) to gatifloxacin and rifampicin; 2 (7%) to ceftazidime, chloramphenicol, and norfloxacin; and 1 (3%) to gentamicin, teicoplanin, and vancomycin (Table 6). Intermediate resistance to amikacin, azithromycin, ceftazidime, ceftriaxone, chloramphenicol, ciprofloxacin, gatifloxacin, gentamicin, teicoplanin, and vancomycin was shown by 1 (3%), 1 (3%), 2 (7%), 3 (10%), 2 (7%), 4 (13%), 3 (10%), 1 (3%), 1 (3%), and 3 (10%) isolates, respectively. Twenty-seven (90%) of the strains tested showed multiple drug resistance (complete or intermediate) to three or more antibiotics. All isolates were completely (100%) sensitive to kanamycin. Additionally, 15 strains were resistant to oxacillin, penicillin, and ampicillin along with other antibiotics and were considered as MRSA strains [9]. All 15 strains further showed their presence on MeReSa Agar and found positive for *mecA* (Fig. 5).

**Table 6:** Antibiotic susceptibility patterns of *S. aureus* isolates

Isolates (n = 30)	Antibiotics																			
	Amikacin	Amoxyclav	Ampicillin	Azithromycin	Ceftazidime	Ceftriaxone	Chloramphenicol	Ciprofloxacin	Gatifloxacin	Gentamicin	Kanamycin	Linezolid	Norfloxacin	Oxacillin	Penicillin	Piperacillin	Rifampcin	Teicoplanin	Tetracyclin	Vancomycin
STRMPL47	R	R	S	S	S	S	S	R	S	S	S	R	S	S	R	R	S	S	S	I
STRMPL23	S	S	R	S	S	S	S	S	S	S	S	S	S	S	R	S	R	S	S	S
STRMBHL57	I	R	R	S	S	S	S	S	S	S	S	R	S	S	R	R	S	S	S	S
STAJM33	R	R	R	S	S	S	S	I	S	R	S	S	S	S	R	R	S	S	S	S
STRMSH56	R	S	R	S	S	S	S	I	I	S	S	S	S	S	R	R	S	S	S	S
STRMSH58	S	S	S	S	S	S	S	S	S	S	S	S	S	S	R	R	S	S	S	S
STRMSH73	R	S	R	R	S	S	S	R	S	S	S	R	S	S	R	S	S	S	S	S
STRMSH74	R	S	R	S	R	S	S	R	S	S	S	S	S	R	R	S	S	S	R	R
STRMSH37	R	S	R	S	S	S	R	S	S	S	S	S	S	R	R	S	S	S	R	S
STRMSH42	S	S	S	S	S	S	S	S	R	S	S	S	S	S	R	S	S	S	S	S
STRMMA20	R	S	R	S	S	S	S	R	R	S	S	S	S	R	R	S	S	S	S	S
STRMMA3	R	S	R	R	I	S	S	R	S	S	S	S	S	R	R	S	S	R	S	S
STRMMA11	R	S	R	R	S	S	S	I	S	S	S	S	S	R	R	S	S	S	S	S
STRMSK96	S	S	S	S	S	S	S	R	S	S	S	S	S	S	R	S	S	I	S	S
STRMSK 98	R	S	R	R	S	S	S	R	S	S	S	S	S	R	R	S	S	S	S	S
STRMSK100	R	S	R	I	S	I	S	R	S	S	S	S	R	S	R	S	S	S	S	S
STRMBP11	R	S	R	S	S	S	S	S	S	S	S	R	R	R	R	S	S	S	S	S
STRMBP12	R	R	R	R	R	S	I	R	I	S	S	S	S	R	R	S	S	S	S	S
STRMJ102	R	S	S	S	S	S	R	R	S	S	S	S	S	S	S	R	S	S	S	I
STRMJ3	R	S	R	R	I	I	S	I	S	S	S	S	S	R	R	S	S	S	R	S
STRMBKN5	R	S	R	R	S	S	S	S	S	S	S	S	S	R	R	S	S	S	S	S
STRMSGNR9	S	S	R	R	S	S	S	S	S	S	S	S	S	S	S	S	S	S	R	S
STRMSGNR19	S	S	R	S	S	I	S	S	S	S	S	S	S	R	R	S	S	S	S	S
STRMSGNR64	S	S	R	S	S	S	S	S	S	S	S	R	S	R	R	S	R	S	S	S
STRMCOR7	S	R	R	S	S	S	S	S	I	S	S	S	S	R	R	S	S	S	S	S
STRMHMO59	R	S	S	S	S	S	S	R	S	I	S	S	S	S	R	S	S	S	S	S
STRMHMO79	S	R	R	S	S	S	S	S	S	S	S	S	S	S	R	S	R	S	S	I
STRMUDZ70	S	R	S	S	S	S	S	S	S	S	S	S	S	S	S	S	S	S	S	S
STRMUDZ2	S	S	R	S	S	S	I	R	R	S	S	S	S	R	R	S	S	S	R	S
STRMUDZ6	S	R	R	R	S	S	S	S	S	S	S	S	S	R	R	R	S	S	S	S
Sensitive	12	22	07	20	26	27	26	14	24	28	30	25	28	15	03	23	27	28	25	26
Intermediate	01	0	0	01	02	03	02	04	03	01	0	0	0	0	0	0	0	01	0	03
Resistant	17	08	23	09	02	0	02	12	03	01	0	05	02	15	27	07	03	01	05	01

## Discussion

In this study, the prevalence, genetic patterns, pathogenic potential, and antibiotic susceptibility of *S. aureus* recovered from 13 districts of Rajasthan, India, were discussed. Our results indicated that 19.84% of milk samples were positive for *S. aureus*, and to the best of our knowledge, this is the first comprehensive study from Rajasthan. Similar findings concerning the prevalence of *S. aureus* in bovine raw milk samples were reported from different sub-parts of India; however, the prevalence rate varied greatly among the studies [30–36]. Of note, moderate prevalence was reported in the present study. We posit that inadequate hygiene and inferior management practices facilitate the entry of microorganisms to udders and concomitantly the shedding of microorganisms into milk.

The phenotypic method is not sufficient for categorizing between coagulase-positive and -negative *S. aureus* isolates [37]. Therefore, we examined the isolates for the *coa* presence by PCR. Nine *coa* PCR types were obtained, signifying considerable variation among the *S. aureus* types and this finding is in agreement with others [14, 23, 38]. The precise reason for this high rate of polymorphism observed among the strains is debatable. However, because of the presence of a series of 81-bp tandem repeats in the 3′-end region of *coa*, the high rate of polymorphism possibly alters the antigenic properties of the gene. Therefore, the gene’s conformity is possibly recognized as the topmost reason behind the failure in the action of neutralizing antibodies [39]. A double band for *coa* was also visualized among three isolates, indicating the existence of distinct allelic forms of *coa*. The appropriate reason behind this distinct *coa* pattern is linked to the production of more than one immunologic form of the coagulase protein by some *S. aureus* isolates [23], although this pattern is rarely observed and only a few authors have reported it [38, 40].

In the present study, the digestion of the different *coa* amplicons with *Alu*I reveals considerable genetic variability as it classifies them into 15 patterns. The findings are similar to those of others [14, 23, 41] who found a considerable degree of variability in *coa* RFLP patterns after digestion with the same enzyme, although the sizes of PCR products obtained here were almost different from those in earlier studies. Besides, the typing method opted for the discrimination was found full-proof as it displayed a fairly good discriminatory power, i.e., of 0.80 and is supported by the findings of Sarvari et al. [15] who obtained a discriminatory index of 0.82 among *S. aureus* isolates.

As mentioned earlier, the study segregated all 30 *S. aureus* isolates into five clusters (A–E), among which patterns I, IV, V, and VI were predominant. Most patterns belonged to clusters A and C. This indicates weak to no heterogeneity in the *Alu*I recognition sites among the isolates of these predominant patterns. Momtaz et al. [14] found that out of 42 *coa*-positive *S. aureus* isolates, 31 (73.8%) belonged to genotype pattern I and 11 (26%) to II. Likewise, in another study [39], pattern I was found predominant. These findings support our results and justify that in a particular geographical region a predominant genotype is present because of prevailing favorable environmental conditions. Besides, the internal resistance of strains toward the host immune system also substantially plays an important role in the strain’s dominance. The predominant isolates from a region have possibly evolved mechanisms to bypass the host phagocytosis than the isolates with rare genotypes [42, 43]. Furthermore, the existence of a similar RFLP pattern for intra-regional strains in genotypes, I, IV, V, VI, VIII, IX, and XIII suggests the possibility of transmission of some strains from one region to another during the transportation of milk.

Next, we tested all isolates for enterotoxin-producing genes and none of them from Ajmer, Jaipur, and Chittorgarh regions scored positive for these genes. In our study, the genes for toxins (*sed*, *see*, *eta*, *etb*, and *tst*) were not amplified in any of the 30 isolates. Enterotoxins contribute to the pathogenesis of *S. aureus* by modulating immunity and susceptibility to antibiotics resulting in the onset of many diseases [44]. The reports concerning the presence of enterotoxin-producing genes (*sea*, *seb*, *sec*, *sed*, and *see*) among *S. aureus* strains isolated from milk of animals with bovine mastitis concur with our findings [44]. Here, we did not find the presence of *eta* and *etb* exfoliative genes, and these results are in agreement with previous studies [11, 44, 45], which inferred that *S. aureus* strains isolated from animals with mastitis were rarely found positive for exfoliative toxins. Similarly, the presence of *tsst*–*1* was not observed in any isolate, although its prevalence in isolates from cattle with mastitis was reported in conjunction with *sec* and *sed* [44].

Most information about the ability of *S. aureus* to form biofilms is restricted to the isolates recovered from clinical studies or medical devices, and insufficient information is available for bovine milk-derived isolates. Therefore, we studied MSCRAMM genes to understand their prevalence. The products of these genes aid the bacterium to adhere to the host components and produce biofilm. Previous studies on MSCRAMM genes in *Enterococcus faecium*, *S. saprophyticus*, and *S. aureus* mention that instead of their active role in biofilm development, some gene products are also involved in pathogenesis. Here, a great diversity in the presence of biofilm-producing genes was observed among the isolates recovered from different regions. The complete dominance was observed for *icaAD* and *eno* responsible for polysaccharide intracellular adhesion and laminin-binding proteins that have a crucial role in bovine mastitis pathogenesis [46]. These findings are in agreement with previous studies [26, 45, 47] that reported higher prevalence rates for these genes. *fnb*A and *fnb*B that are usually associated with invasive diseases [48] were also found abundantly (97 and 80%, respectively) among the isolates in this study; these rates are comparable to those in other studies [20, 26]. Similarly, *clf*B encoding an Fn-binding protein that facilitates *S. aureus* to establish nasal colonization [49] was also present in most (93%) strains. Proteins EbpS, SasG, and SasC, which play a considerable role in maintaining cell density, biofilm accumulation, and intercellular adhesion, were present in 90, 80, and 53% of isolates, respectively. These findings are comparable and in complete agreement with the findings of Tang et al. [26] who noticed a high prevalence of these genes. Another collagen-binding protein, Cna, which has been associated with virulence potential in rabbit models [48], was identified in 77% of isolates; this value is comparably higher than that reported by Pereyra et al. [50] but lower than others [20, 26]. Gene *bbp*, which is often found in the clinical strains of *S. aureus* and linked with bone infections, was only typed in a few (6.6%) isolates. The result is in accordance with the findings of Kot et al. [51] and Puacz et al. [52] who detected the presence of *bbp* in a few isolates of mastitis origin. Gene *bap*, whose protein was probably the first protein elucidated to have a role in biofilm formation in *S. aureus* and associated with chronic bovine mastitis, was present in 7% of isolates. This finding is consistent with the observations of Darwish and Asfour [45], who observed the presence of *bap* in 2.5 and 4.4% of *S. aureus* and coagulase-negative *S. aureus* isolates, respectively. Another gene *fib*, elucidated to have a crucial role in binding to extracellular matrix fibrinogen, was also detected in 10% of isolates. This finding is different from the findings of Pereyra et al. [50] and Zuniga et al. [53] who reported a higher prevalence of 90 and 71.7%, respectively. Altogether, our results suggest that most strains had the biofilm-forming ability, which may facilitate in establishing intramammary gland infections in cattle. Moreover, this property varies from strain to strain and depends on the genetic composition and geographical evolution.

In the present study, the observed antibiotic resistance level was relatively high (90%) and two strains (STRMSH74 and STRMBP12) from Sirohi and Bharatpur, respectively, were found to be most resistant. MRSA-positive strains were observed from each area except for Pali, Bhilwara, and Hanumangarh; this finding is in agreement with studies reporting varied MRSA prevalence in bovine milk [9, 43, 54]. No similarities in resistance patterns were observed among the strains from different regions except for one strain each from Jaipur (STRMJ3) and Bikaner (STRMBKN5). The inconsistencies in the resistance profile observed might be due to the excessive, indiscriminate use of a particular antibiotic in each geographical region for combating infections. This fact was established by similar findings in studies undertaken in the United States and European countries, where a lower resistance profile among *S. aureus* isolates was noticed because of stricter use of antibiotics in veterinary practice [54]. In Rajasthan, β-lactams, macrolides, aminopenicillins, cephalosporins, aminoglycosides, fluoroquinolones, tetracyclines, and amphenicols are frequently used in veterinary clinical practices. However, resistance toward the less commonly used antibiotics such as vancomycin, teicoplanin, and rifampicin in veterinary practice is not fully understood. This is possibly due to the contamination from human handlers [18], as strains with rare and high antibiogram patterns were generally isolated from hospital settings because of a high antimicrobial pressure [54]. Earlier, vancomycin-resistant enterococci strains were isolated from tertiary care hospitals in Rajasthan [55, 56], and it was proved that the gene cluster VanA was transferable from *E. faecalis* to *S. aureus* [57]. Similarly, teicoplanin-resistant *S. aureus* strains were reported from a hospital in Jaipur, Rajasthan [57]. Thus, considerable attention is needed for treating the vancomycin-resistant MRSA-induced infection. Based on our results, all strains were sensitive to kanamycin, and it could be the drug of choice to treat *S. aureus* mediated infections.

Overall, our results clearly indicate that bovine raw milk samples collected from different regions of Rajasthan were contaminated with coagulase-positive strains of *S. aureus.* Considerable variability in *coa* was observed, and genotypes I, IV, V, and VI were predominant. The close similarity in the RFLP pattern among the isolates of different regions suggested the transmission of isolates from one place to another by various means. The presence of enterotoxin and biofilm-producing genes among these strains poses a potential public health risk, as these strains may be implicated in milk-borne intoxications. Furthermore, the presence of multidrug-resistant and MRSA strains indicates the improper use of antibiotics for mastitis control and is a growing health concern globally. However, all strains were found susceptible to kanamycin, and it can be referred to as a drug of choice in case of mastitis infections in the regions included in the study. The information generated here might be useful for concerned veterinarians in improving cattle health and designing strategies for better and safe milk production. This will not only lower the risk of associated food poisoning but also prevent the spread of antibiotic resistance in the regions. Future research is warranted to establish the relationship between the presence of these strains in milk and their ability to cause infections in humans.
